# The Experiences of Family Members of Patients Discharged from Intensive Care Unit: A Systematic Review of Qualitative Studies

**DOI:** 10.3390/nursrep14020113

**Published:** 2024-06-14

**Authors:** Benedetta Basso, Sebastiano Fogolin, Matteo Danielis, Elisa Mattiussi

**Affiliations:** 1 School of Nursing, Department of Medical Sciences, University of Udine, Viale Ungheria 20, 33100 Udine, Italy; basso.benedetta@spes.uniud.it (B.B.); sebastiano.fogolin@uniud.it (S.F.); elisa.mattiussi@uniud.it (E.M.); 2 Laboratory of Studies and Evidence Based Nursing, Department of Cardiac, Thoracic, Vascular Sciences and Public Health, University of Padua, Via Loredan 18, 35131 Padova, Italy

**Keywords:** family members, experience, perceptions, intensive care unit, discharge, qualitative

## Abstract

Background: Improving the understanding of the post-discharge experiences of family members after their loved ones leave the Intensive Care Unit (ICU) is essential for developing effective follow-up strategies. These strategies are crucial for mitigating potential negative outcomes for both patients and their families. The aim of this study was to explore the lived experiences of family members after the discharge of their loved ones from the ICU. Methods: In September 2023, we conducted a systematic search of qualitative studies across the following databases: CINAHL, MEDLINE, Scopus and Web of Science. The Preferred Reporting Items for Systematic Reviews and Meta-Analysis (PRISMA) was used to guide this review. Results: Eight articles met the inclusion criteria. Four themes were identified following evidence synthesis: (1) grappling with a weighty burden; (2) recognizing and confronting adversities along the way; (3) seeking support beyond one’s own resources; and (4) addressing comprehensive care requirements. Conclusions: Family members face significant psychological and physical challenges while caring for their loved ones recovering from an ICU stay. Adequate formal and informal help is imperative to provide support both during hospitalization and after discharge. A refined understanding of the distinct requirements and experiences of family members can serve as a strategic framework for informing educational interventions and follow-up programs during the transition from hospital settings to community-based care. This study was not registered.

## 1. Introduction

Intensive Care Unit (ICU) admission is a stressful time for both patients and their families [1,2]. During an emergency, family members may not have the adequate time to make care decisions that respect the patient’s wishes and dignity [3]. During the ICU stay, family members serve as proxies for patients, as the severity of a patient’s clinical condition may hinder their ability to participate in medical decisions [4]. The experience of intensive care admissions and the challenges faced by family members after their loved ones are discharged from the ICU are associated with the emergence of predominantly negative psychological, physical, and cognitive sequelae, falling under the definition of Post Intensive Care Syndrome—Family (PICS-F) [1,5,6]. As defined by the Society for Critical Care Medicine, PICS-F describes new or worsened physical, cognitive, or mental impairments that occur after critical illness and persist beyond acute care hospitalization [6,7]. However, despite the growing number of ICU admissions, the psychological consequences for family members are not yet fully understood and adequately addressed, especially after returning home [8]. Going back to everyday life implies adapting to a new lifestyle, including increasing awareness of the patient’s limitations and reorganizing the home environment. The difficulties and challenges faced by family members after patients’ discharge from the ICU are well-documented in the literature, but may vary depending on the historical and socio-economic context, and the characteristics of the population [9]. Indeed, many family members feel lonely, disoriented, and confused in managing the physical and psychological needs of a patient who suffered a critical illness. In the first few months after discharge, family members face new challenges that inevitably have an impact on their lives [10]. However, identifying the risk factors for PICS-F is challenging, which makes it difficult to detect high-risk families and provide targeted interventions. There is a lack of awareness among primary care physicians and hospital professionals regarding the psychological experiences of family members of ICU survivors [11]. A deeper understanding of family experiences and their real needs can contribute to strengthening family-centered care and reducing the symptoms of PICS-F in the long term. The aim of this study was to explore the lived experiences of family members following the discharge of their loved ones from the ICU.

## 2. Materials and Methods

### 2.1. Study Design

The study design is a systematic review of qualitative studies with a meta-synthesis. In this study, we chose to employ a thematic synthesis as recommended by Thomas and Harden [12]. It consists of extracting relevant text from the results section of the included studies, coding it inductively and line-by-line, and then organizing the codes into related categories to form descriptive themes.

### 2.2. Study Selection

We utilized the Population, Exposure and Outcome (PEO) mnemonic [13] for qualitative research to guide and organize the inquiries. The PEO model was P (family of patients discharged from the ICU), E (daily life experience) and O (experiences of family members after discharge from the ICU).

A thorough search of the literature was conducted in September 2023 (last search: 30 September 2023), using a combination of Medical Subject Headings (MeSH) terms and keywords such as: “Family”, “Experience”, “Intensive Care Units”, “Discharge” and “Qualitative Research”. For the purpose of this review, we define “family” as encompassing the patient’s spouse, partner, individuals of a blood relation, next of kin, caregivers, and/or those with whom the patient predominantly spends their time.

All MeSH terms and free text words have been combined into search strings using the Boolean operators “AND” or “OR”. The search strategy included the following databases: Cumulative Index to Nursing and Allied Health Literature (CINAHL), MEDLINE (PubMed), Scopus and Web of Science.

### 2.3. Criteria for Inclusion

**Figure 1:**
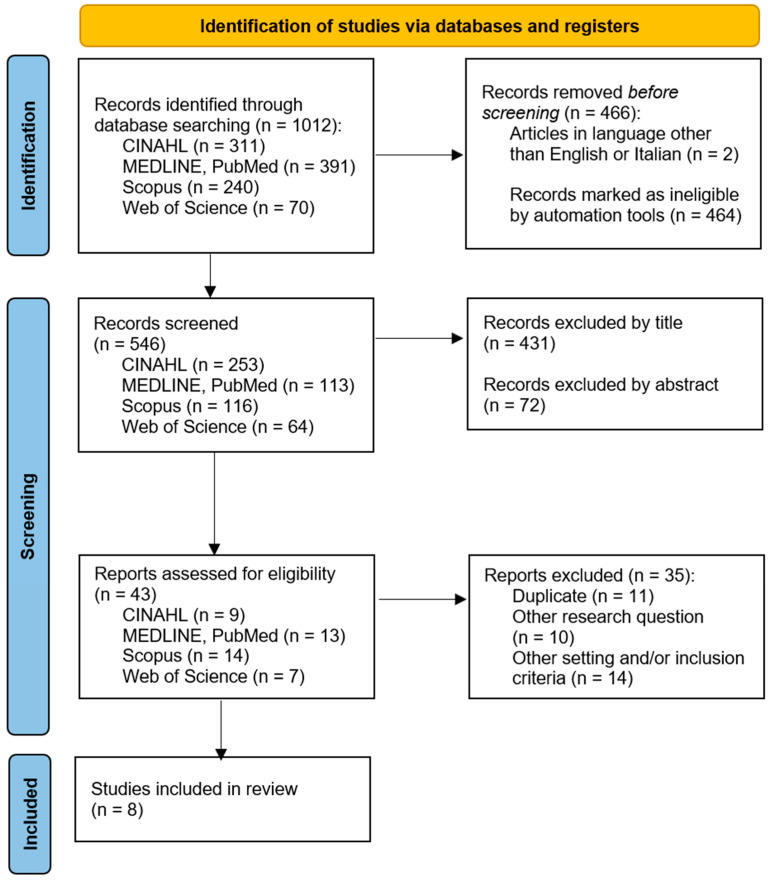
Study selection process (PRISMA) flowchart.

The review followed the guidelines outlined in the Preferred Reporting Items for Systematic Reviews and Meta-Analyses (PRISMA) Statement [14], which facilitated the identification, screening, and confirmation of eligibility for including primary literature, as shown in Figure 1 below. Articles were considered for inclusion based on the following criteria: qualitative studies conducted through semi-structured interviews with both open and closed questions that focused on the perceptions, experiences, opinions, challenges, and feelings of family members after a loved one was discharged from the ICU. Studies utilizing phenomenological, hermeneutic, and thematic analysis approaches were accepted. Additionally, studies sampling patients were included if they explored the experiences of family members.

Database searches were limited to studies published between 2013 and 2023. This time limit was selected to include the most recent surveys, because the difficulties and challenges faced by family members after discharge from the ICU may vary depending on the historical and socio-economic context.

Exclusion criteria involved studies with patients under the age of 18; those not reporting on the experiences of family members; or those conducted in clinical settings other than the ICU or pediatric/neonatal ICU settings. There were no restrictions on the country of publication, but only articles published in English were selected.

The search yielded 1012 results. At this stage, we implemented Rayyan (http://rayyan.qcri.org, (accessed on 10 October 2023)), a free web and mobile app, that helped expedite the initial identification of studies using a process of semi-automation. Prior to screening, two articles were removed due to being in a language other than English/Italian, and 464 were flagged as ineligible by Rayyan. Out of the 546 screened, 43 articles were assessed for eligibility; of these, 35 were excluded for the following reasons: 11 were duplicates, 10 were deemed irrelevant to the research question, and 14 were not relevant according to the inclusion or exclusion criteria or the setting. Finally, eight studies that fully satisfied all inclusion criteria were identified. The titles, abstracts, and full texts of the selected papers were assessed by two independent reviewers against the inclusion criteria (EM, BB). After that, all eight studies were included in this review.

### 2.4. Quality Appraisal

The methodological quality of the included studies was assessed using the Critical Appraisal Screening Programme for qualitative studies [15]. This tool is aimed at evaluating the quality of ten methodological domains, and each is reflected in an item that can be scored as “Yes” (Y), “No” (N), or “Cannot tell” (U), depending on whether they have been described appropriately in the full text of the article; higher scores indicate a high study quality. According to previous research, [16] the authors considered the quality levels to be low (CASP 0–5.5), medium (CASP 6–8.5), and high (CASP 9–10) according to the total scores obtained. A breakdown of the completed checklist for each paper is presented in Table 1.

### 2.5. Data Extraction

Two researchers (ME, BB) used a standardized Microsoft Excel^®^ spreadsheet to extract data from selected studies, as shown in Table 2.

The following data were obtained: (a) Research Title and Authors; (b) Types of Research; (c) Setting and Country of Research; (d) Aim of Research; (e) Data Collection Methods; and (f) Main Findings.

### 2.6. Characteristics of Included Studies

The records included in this review all have a qualitative study design. Three studies were published in the triennium 2013–2016, one study in the triennium 2017–2019, and four studies in the triennium 2020–2023. The majority of studies were published in Europe (Denmark, Italy, Norway, Netherlands and Sweden), while the remaining two were released in North America (Canada and USA). The combined total of participants in the eight studies included in this review was 98: 71 (72.5%) are females, while 27 (27.5%) are males. In all studies, except for two, the degree of kinship of the family members recruited for the interviews in each individual study is explicitly stated (Table 3). Although all patients and their families experienced admission to the ICU, the specialization of the operating unit varied. Specifically, patients were admitted to general, neurosurgical, medical, and medical-surgical ICUs. All studies (*n* = 8) used semi-structured qualitative interviews that took place after the patient’s discharge from ICU. The interviews were conducted by telephone or face-to-face in different settings: either inside the hospital (not in the ICU) or outside (at the homes of discharged patients’ relatives, in a library, or in locations chosen by the family members). The interval for the follow-up interviews with family members, which were conducted after a patient was discharged from the ICU, ranged from a minimum of 14 days to a maximum of three years.

## 3. Results

Four main themes arose from the studies: (1) grappling with a weighty burden; (2) recognizing and confronting adversities along the way; (3) seeking support beyond one’s own resources; and (4) addressing comprehensive care requirements through multi-faceted intervention. The whole inductive analysis can be found in Supplementary Table S1.

### 3.1. Theme 1: Grappling with a Weighty Burden

The relatives of a critically ill patient discharged from hospital face many difficulties that burden their everyday lives. Overall, the included literature describes the maladaptation to the caregiving role. Many negative feelings are perceived while transforming their role as a family member into that of a caregiver. The prevalent feeling is of being overwhelmed. The relatives describe the important tasks to accomplish, frequently done without any help: “*It takes so much time. I do it every time. I had to go to the airport the other day, you know. Then, we needed some milk. Then, the car needs a check-up. I have to go again… every time. Food… I have to shop and cook each day.*” [17]. The patient himself represents a source of hard work: “*Oh, I had no idea what I was in for [when my son came home] … I don’t think I slept two hours the first week. That’s up and down the stairs; he was so much work*” [18]. When the patient arrives home, relatives feel worried about the caring duties: “*Coming home is a little scary to me. I worry about taking care of him… what it’s gonna involve.*” [20]; but also, for what can happen daily: “*It’s still a worry-when I wake up in the morning*” [18]. The huge work done by the parents leaves them exhausted physically and emotionally: “*It seems I’m even more tired now than I was when she was sick and right out of the hospital. I don’t know whether it’s just catching up with me or not, but I’m mentally and emotionally exhausted… Everything just seems like a struggle lately.*” [20]. Along with this important emotion, relatives feel lost: they perceive being left alone, “*Left without a lifeline*” [23], at risk of making mistakes, “*It’s easy to make mistakes, and nobody else was there….*” [17]. Moreover, this perception of being left alone is accompanied by a sensation of frustration when they understand that nobody is giving suggestions to deal with the patient and its disease: “*No one can tell us how to increase that activity level appropriately; it’s really just trial and error, so it’s a little frustrating.*” [20]. The sense of frustration also arises when all the strategies applied to care for the patient do not seem to improve their clinical situation: “*It was frustrating to discover that things got lost in the system. I discovered that there were other options, but they were dead ends as well*” [17].

In some reports, relatives describe their perceived weight of responsibility. Many activities represent a commitment in terms of responsibility, “*Suddenly I got a lot of responsibilities, things I had never tried, and I just didn’t know what to do.*” [17], but also their own role is part of this responsibility, “*I feared I pass away, but the real fear is: how will my wife save herself then? As a matter of fact, she can’t miss [function without] me.*” [22]. Becoming a caregiver involves leading many roles: “*I have a different role here. I have to fill many roles at the same time and be ‘husband and wife, caregiver or lover,’ or whatever we call it…*” [17]. All of these circumstances challenge relatives in their social relationships, both in terms of their shared relationships with others, “*I was not [comfortable going] out in public. I had tried a couple of times at the store, but then someone came to me, people approached me, and I could not handle that they had asked me about things…*” [19], and the interaction of others in their private lives “*After this happened to my husband, I had a fight with a lot of people, as I thought they interfered with things they shouldn’t interfere with.*” [22].

### 3.2. Theme 2: Recognizing and Confronting Adversities along the Way

Through their experience at home as caregivers, relatives recognize adversity over time. The different challenges encountered characterize their care journey both positively and negatively. The first challenge is having to provide health care for one’s loved one while managing medical complexities. Relatives seek a lot of information to guide their own care activities, “*I received quite a lot of information; I think mainly because I was very persistent. I was always there asking questions and then if they told me anything, I basically went back to the computer and researched it […] because the information that was passed on to me was, at that time, too much to take in because I had no medical knowledge.*” [18], also recognizing that the skills required are predominantly health-related, “*That day I just didn’t know what to do…. I finally thought: What should I do? I am not a nurse.”* [17]. A second challenge is the workload of care: “*I had to take time of work to assist her*” [10]. Not being aware of the patient’s prognosis makes the course of treatment more uncertain, “*No one seems to know how long his condition is going to be the way it is or if it is ever going to be any different, if he’s ever going to get better, or if he’s just going to stay the same.*” [20], and triggers a sense of uncertainty: “*I just hope the lung infection doesn’t come back again…he wouldn’t survive another illness*” [10].

Despite the complexities of the journey, relatives are able to cope with their demanding situation, and although some challenges represent uncertainties or workloads, relatives also confront the knowledge that they need to let things go at their own pace, “*You lean back and say: Okay, it will just have to go as it goes. You just give up and let things go at their pace.*” [17], and that the new experience is part of life, “*The experience is part of who I am; in this way, I will continue my life*” [23]. Moreover, in spite of the feelings of fatigue described in acting out their role as caregiver, they also feel strong in that position, “*I am not used to asking for help. You manage on your own. I discovered an inner strength, and I am stronger than I thought*” [19], even focusing on their own capabilities, “*I have been good at handling things myself, and, of course, having my children around all the time. So, I had someone to talk to. But not too much; I felt I had to do it myself… so I have been working on that…*” [19]. Recognizing important values in life and in lived experience represents a form of support, “*I didn’t have anything to do with the church. However, I’ve been thinking differently about this. It helped me in a positive way.*” [21], and awareness for relatives in dealing with the new reality: “*This is how life is. It has a beginning and it has an end. And you can’t make the end worse than it has to be. Instead, you have to think back on the good things we have experienced together, all the trips we have made. That’s how you need to think*” [21]. Recognizing important values also enables relatives to make choices with respect to their role, “*Life-threatening experiences changes your perspective. The friends who stick around and listen, you hold onto. Those who don’t, you end*” [23]; “*…thus you end up neglecting much of your own feelings; what is bothering you is neglected. I have put it away, and I have to deal with it little by little*” [19].

### 3.3. Theme 3: Seeking Support beyond One’s Own Resources

The lived experience of family members emerges as complex, exhausting, and challenging, yet family members describe seeking support beyond their own personal resources.

Family members describe both professional and peer support received. They recognize the help provided by the healthcare system, “*At home, we are followed by home care, a nurse visits us. If there are any critical issues, we ask her*” [10], and recognize how important it is: “*The home care nurse…, she was phenomenal. … she was just tremendous. She just walked us through everything. I felt really good while she was there, and after she left, I knew everything would be okay.*” [18]. Even if in some reports experiences with the health care system are described positively, there are also negative experiences: “*I feel that there was a two-week delay for his rehabilitation to start. And the reason I’m emphasizing the delay is because two weeks after an ICU stay for a survivor is a long, long time.*” [18]. Another form of help that is appreciated is the support provided by friends and relatives, “*Both family and friends have been very good at visiting, writing, and calling.*” [17], and the comparison with one’s peers, “*Listening to people sharing their struggles with everyday life validated my experiences. They were finally real!*” [23].

Despite the support received, family members describe their essential needs for caregiving. In several reports, the need for assistance is represented, both short-term, “*I finally called the doctor and asked him to come as fast as possible.*” [17], and long-term, “*I think probably [would have liked to receive more contact] with the health care system, because you’re not quite sure as to how your recovery period is going.*” [18]. The need to be able to rely on a case manager is specified: “*…there isn’t someone managing the whole thing, managing all the components of his life… like how to fully recover, there should be someone managing the whole case…*” [18]. Psychological support is also considered essential for themselves, “*Actually, I considered whether I needed help from a psychologist to clear up my thoughts, [so] that I’m not as mean when I’m upset*” [19], and for the patient, “*They recommended that she see a therapist, and we thought that was dumb. But now I wished she would have done it earlier…. I think she is even more depressed*” [20].

### 3.4. Theme 4: Addressing Comprehensive Care Requirements

Caring for a critically ill patient sent home requires a targeted provision of care. In two reports, the need to prepare the home for patient reception is described: “It was a little bit hard at the beginning because I had to renovate and change some things at home. Put in some technical aids. Our old house is definitely not suited for a handicapped person…” [21]. The family members also describe the activities performed as caregivers. The need for patient supervision is described in multiple reports, “*… the first thing I do is see if she’s alright, I go across to the bed, and she’s lying there sound asleep …*” [18]. The need for supervision can vary as one’s loved one’s condition changes: “*The three of us in the family are home and observe her all the time; if she has a bad day, worse than usual, we are much more alert.*” [17]. Caring for your loved one also means supporting them where their physical condition is limited, “*My main job right now is to help him pace himself appropriately and to help him when he doesn’t.*” [20], and when they need to be stimulated, “*I couldn’t just sit and watch how he had fought at the hospital and then just faded away. So, I said that either he would pull himself together and take a walk, or I would go to the summer house and he would have to manage on his own*” [17]. In one report, caregivers also face the need to tell their loved ones about the care journey they faced, “*I don’t want to be a nag, but I want to try to give him a realistic picture of where he is at and where he is going… I am glad he listens, and hopefully that will help prevent him from having unrealistic expectations and getting frustrated as he goes along over the next few months.*” [20]. Not in all descriptions was this activity considered easy to be conducted and effective for the patient: “*I don’t want to remind him how bad he was…. I find it hard for him to talk about it sometimes. I don’t know if it makes things worse when we talk about it because it reminds him of how weak he was.*” [20].

Care delivered at home also depends on the patient’s dependence. For the family members, the patient’s participation is considered to be fundamental to the recovery path, “*I couldn’t get him to go to rehab. He just sat on the couch and stared out the window…. But then he got up and walked to the beach and back. The day after, he came along with me twice*” [17]. In some cases, the patient fails to participate in the care pathway “*The most difficult thing is seeing him not beg able to deal with it. He just breaks down, and he just sits there and cries because he does not know what to do. And it is sort of hard to see him when I know he is such a strong guy.*” [20]. The course of the disease and its outcomes over time also affect the patient’s level of dependence. Some family members describe the potential impact of the patient’s clinical progress: “*He doesn’t walk, he doesn’t move by himself, he can’t even sign.*” [10]; some others describe different kinds of changes that have an impact on the continuity of care, “*He says that he hasn’t suffered permanent damage, but it just isn’t true. He has lots of problems, but not physical ones. I mean, invisible things that have changed.*” [17].

## 4. Discussion

This systematic review offers a novel interpretation of the experiences lived by family members following the discharge of their loved ones from the ICU. The return home of patients after hospitalization in ICUs represents a real challenge for caregivers who are faced with a high burden of care due, on the one hand, to the weight of different responsibilities with respect to the tasks required and the different roles exercised; on the other hand, to the negative feelings such as anger, fear, and frustration that characterize a condition of maladaptation to the role of caregiver. From this point of view, the results of this study contribute to improving the description of PICS by confirming the main characteristics reported in various studies [7,8].

Some strategies resulting from the combination of post-discharge follow-up programs, the caregiver’s diary, and clear and comprehensive information have already demonstrated their potential to reduce post-traumatic stress symptomatology in family members, alleviating the discomfort related to the presence of PICS [24]. Therefore, the early identification of PICS signs and symptoms through systematic follow-up strategies, such as individual consultations by dedicated health care professionals, psychological support, participation in self-help groups, or the use of diaries [25], could improve caregivers’ ability to cope. In particular, diaries help caregivers express their emotional experiences, strengthening their ability to cope with the challenges they will encounter during the caring process.

The care burden of family members, moreover, increases according to the degree of dependence that the patient presents at home, not only physically but also psychologically. The patient’s disability after admission to the ICU and the need for care involve a change in family roles and responsibilities with work and financial issues, leading to a disruption in the functioning and integrity of the family, with an interruption of the home routine [6]. It is crucial that caregivers do not feel alone, and that they can count on positive support from the health care system but also from the support provided by friends and relatives. In this regard, a further strategy is the involvement of the family in the intensive care unit, which can reduce the sense of anxiety and fear resulting from not feeling prepared to take on the role of caregiver. The early care of the patient and family in the ICU is an effective tool in recognizing those at greater risk of developing complications during and after hospitalization, in order to initiate preventive measures to ensure the continuity of care at home as well [26,27]. The implementation of follow-up programs not only helps to decrease anxiety and fear of the unknown but also to reduce the feeling of loneliness during the transition home. Similarly, these programs improve the understanding of the daily challenges patients deal with at home.

Future research should focus on the development of a screening tool aimed at the early identification of family caregivers at risk of developing health problems, providing them with timely support [6,28]. Primary healthcare professionals should have a comprehensive understanding of caregiving-related stress and assess its impact on caregivers’ well-being [11]. Needham et al.’s study asserts that guidelines have been developed for the overall assessment of caregivers [5]. However, it is necessary to create more specific guidelines for family members going through the ICU experience; these guidelines could assist primary care physicians and nurses in systematically collecting information on the psychosocial aspects concerning the caregiver. In primary care settings, the healthcare professionals should play a role in identifying and coordinating the treatment of these symptoms, referring caregivers, for example, to a social worker or psychotherapist. Recent studies highlight that many primary care physicians may not be familiar with the specific needs of these caregivers [11]. To further improve the understanding of the different challenges and needs of family caregivers, it is necessary to understand how the diverse experiences of post-ICU family members may vary based on ethnic, socioeconomic, and cultural contexts. Additionally, the role of society in supporting informal caregivers after hospital discharge and the identification of specific relevant interventions are other areas that require further attention [17]. Moreover, the number of interventions and studies aimed at reducing or mitigating the consequences of caregiver burden is limited; thus, further research is needed in this regard.

This qualitative synthesis has some limitations. First, we acknowledge the limitations of a systematic review of qualitative studies in fully addressing the complexity of post-ICU family experiences. Second, we recognize the heterogeneity of the countries in which the studies included in this meta-synthesis were conducted. The absence of studies from low- and middle-income countries underscores the gap in the research landscape. This omission underscores the diversity of healthcare provision systems and the need to explore nuanced social and cultural contexts within these regions. Although the results that emerged in this meta-synthesis are consistent with those already present in the literature, there are several factors (ethnic, socioeconomic, cultural, clinical, and organizational) that can significantly influence how families experience their loved one’s return home from the ICU. While the studies included in our analysis only report data on biological sex and relationship degree, this limitation may hinder a comprehensive understanding of gender-related differences. Nonetheless, it is notable that the majority of informal caregivers are women, a trend linked to potentially significant impacts on their physical and mental well-being [29]. This observation suggests societal expectations that place greater emotional responsibility on women within relationships. Despite the absence of explicit gender data, these findings underscore the importance of considering gender dynamics in caregiving research and policy initiatives. Lastly, half of the studies were conducted before the COVID-19 pandemic, while the other half were published subsequently. This could lead to a variation in the experiences, difficulties, and challenges encountered by family members after discharge from the ICU.

## 5. Conclusions

This study explored the experiences of 98 family members of patients discharged from the ICU, highlighting the daily routine care, emotions, challenges, and needs experienced. The findings highlight the need for the support that a critical patient home discharge pathway requires in the long term. In general, the literature indicates that actively involving family members during an ICU stay can improve clinical outcomes and family satisfaction. However, many family members feel unprepared to manage care at home and may develop long-term psychological issues. It is essential to provide training and early support to family members, especially with regard to the post-discharge period. Post-ICU care models, such as follow-up clinics, can be crucial in providing long-term multidisciplinary support and need to work closely with community care to tailor the assistance in the long-term. Healthcare professionals need to be educated on assessing and meeting the needs of family members, improving communication, and providing detailed information and resources to manage the transition at home. 

## Figures and Tables

**Table 1:** The Critical Appraisal Screening Programme for included studies.

	Ågård et al., 2015 [17]	Czerwonka et al., 2015 [18]	Frivold et al., 2016 [19]	Choi et al., 2018 [20]	Nelderup et al., 2020 [21]	Van Sleeuwen et al., 2020 [22]	Danielis et al., 2022 [10]	Vester et al., 2022 [23]
Section A								
Q1. Was there a clear statement of the aims of the research?	Yes	Yes	Yes	Yes	Yes	Yes	Yes	Yes
Q2. Is a qualitative methodology appropriate?	Yes	Yes	Yes	Yes	Yes	Yes	Yes	Yes
Q3. Was the research design appropriate to address the aims of the research?	Yes	Yes	Yes	Yes	Yes	Yes	Yes	Yes
Q4. Was the recruitment strategy appropriate to meet the aims of the research?	Yes	Yes	Yes	Yes	Yes	Yes	Yes	Yes
Q5. Was the data collected in away that addressed the research issue?	Yes	Yes	Yes	Yes	Yes	Yes	Yes	Yes
Q6. Was the relationship between researcher and participants adequately considered?	No	No	No	No	No	No	Yes	No
Section B								
Q7. Were ethical issues taken in consideration?	Yes	No	Yes	No	Yes	Yes	Yes	Yes
Q8. Was the data analysis sufficiently rigorous?	Yes	Yes	Yes	Yes	Yes	Yes	Yes	Yes
Q9. Is there a clear statement of findings?	Yes	Yes	Yes	Yes	Yes	Yes	Yes	Yes
Section C								
Q10. How valuable is the research?	Yes	Yes	Yes	Yes	Yes	Yes	Yes	Yes
Total Score	10	8	9	8	9	9	10	9

NB: This table is an adaptation of the Critical Appraisals Skills Programme (CASP) Qualitative Checklist (2018).

**Table 2:** Summary of included studies.

Title, Authors, Publication Year	Design	Setting and Country	Aim (s)	Data Collection Methods	Main Findings
From spouse to caregiver and back: a grounded theory study of post-intensive care unit spousal caregiving Agard et al., 2015 [17]	Qualitative study based on the Grounded Theory methodology	General and Neurosurgical ICU, Denmark	To explore the challenges facing spouses of ICU survivors; to describe and explain their concerns and caregiving strategies during the first 12 months post-ICU discharge	Semi- structured interviews	Spouses have a crucial and multifaceted role in the recovery process after leaving the ICU; they have lived through the transition of their role from spouse to caregiver and back. Hospital staff, rehabilitation specialists, and primary care providers should recognize the significant contribution of spouses
Changing support needs of survivors of complex critical illness and their family caregivers across the care continuum: A qualitative pilot study of Towards RECOVER Czerwonka et al., 2015 [18]	Qualitative study using the Timing It Right framework	University-affiliated medical-surgical ICUs, Canada	To explore participants’ experiences and needs for information, emotional support, and training at 3, 6, 12, and 24 months after intensive care unit (ICU) discharge	Semi- structured interviews	Interventions targeting improved family outcomes after critical illness should consider the changing support requirements of both survivors and caregivers as they progress through the illness and recovery process. Early intervention and transparent communication regarding care transitions and recovery can help alleviate uncertainties for all involved. Ongoing family-centered follow-up programs have the potential to assist caregivers in managing their perceived caregiving duties
Family members’ lived experiences of everyday life after intensive care treatment of a loved one: a phenomenological hermeneutical study Frivold et al., 2016 [19]	Phenomenological hermeneutical method inspired by Lindseth and Norberg	General and Medical ICU, Norway	To illuminate relatives’ experiences of everyday life after a loved one’s stay in an intensive care unit (ICU)	Semi- structured interviews.	Nursing education could prioritize the importance of communication and personalized support, aiding family members in coping during the patient’s hospitalization and fostering a sense of resilience upon returning home. After coming back home, it is crucial for family members to retain self-control and adjust to these changes for future readiness. They manage by tapping into their personal resources and relying on support from others. Additionally, some may require additional follow-up from the intensive care unit staff
Home discharge following critical illness: A qualitative analysis of family caregiver experience Choi et al., 2018 [20]	Descriptive qualitative study with a content analysis	General ICU, USA (Pittsburgh)	To describe the varying challenges and needs of family caregivers of ICU survivors related to patients’ home discharge	Semi- structured interviews.	Family caregivers of ICU survivors require knowledge and expertise to assist in managing patients’ care requirements, aligning expectations with the actual progress of patients, and addressing the health needs of caregivers themselves.
Health problems among family caregivers of former intensive care unit (ICU) patients: an interview study Van Sleeuwen et al., 2020 [22]	Exploratory qualitative study according to Braun and Clarke’s six- phases	General ICU, Netherlands	To explore health problems in family caregivers of former ICU patients and the consequences in their daily lives	Semi- structured interviews.	Caregivers continue to face various health issues, persisting long after their loved ones are discharged from the ICU. It is crucial for healthcare providers to prioritize the health of not just ICU patients but also their caregivers. Identifying and addressing caregivers’ health concerns at an earlier stage is essential to providing them with the necessary care and support
Experiences of partners of intensive care survivors and their need for support after intensive care Nelderup et al., 2020 [21]	Qualitative descriptive study with a content analysis	General ICU, Sweden	To explore the experiences of partners of intensive care survivors and their need for support after ICU	Semi- structured interviews.	Partners require comprehensive and ongoing support from healthcare professionals and others throughout and after post-intensive care periods. While intensive care can often engender feelings of chaos for partners, strengthening family relationships and providing appropriate comforting support can mitigate this chaos and pave the way for a smoother recovery journey, fostering a more positive outlook on the future
Patients’ and relatives’ experiences of post-ICU everyday life: A qualitative study Vester et al., 2022 [23]	Qualitative study within the phenomenological-hermeneutic tradition	Multidisciplinary ICU, Denmark	To explore patients’ and relatives’ experiences of everyday life after critical illness	Semi- structured interviews.	The research highlights the significance of broadening the recognized aspects of PICS to encompass a social dimension, facilitating family-centered care within and outside the ICU. Additionally, it underscores the importance of developing tailored rehabilitation approaches to address the diverse health needs of both patients and their relatives
Experience of relatives in the first three months after a non-COVID-19 Intensive Care Unit discharge: a qualitative study Danielis et al., 2022 [10]	Descriptive qualitative study with a thematic analysis	General ICU, Italy	To explore and describe the experiences of a relative who has been facing day-to-day life during the first three months after a non-COVID-19 ICU discharge	Semi- structured interviews.	Upon discharge, family members confronted constraints in community services, compelling them to seek supplementary assistance from private healthcare providers. Moreover, changes in the patient’s treatment plan intensified specific caregiving challenges, resulting in a sense of isolation. Relatives encountered a twofold limitation in opportunities, both within the hospital, with restricted involvement and limited access to ICU accessibility, and at home, concerning formal and informal care alternatives

**Table 3:** Studies’ participants’ characteristics.

	Gender		Relationship Degree	Length of Stay in ICU Days Range (Mean)	Timing of Follow-Up Interview Months
	M	F			
Ågård et al., 2015 [17]	7	11	11 wives, 7 husbands	5–74	3–12
Czerwonka et al., 2015 [18]	1	6	U	10–64 (29)	3–6–12–24
Frivold et al., 2016 [19]	6	7	1 son, 6 wives, 3 husbands, 1 brother, 1 mother, and 1 grandson	2–42	3–13
Choi et al., 2018 [20]	4	16	13 spouses or significant other, 3 adult child, and 4 parents or siblings	5–39 (23)	0.5–2–4
Van Sleeuwen et al., 2020 [22]	3	10	U	>5 (4.5)	3–36
Nelderup et al., 2020 [21]	2	4	4 wives, 2 husbands	10–69	6–10
Vester et al., 2022 [23]	1	6	4 wives, 2 mothers and 1 husband	1–14	U
Danielis et al., 2022 [10]	3	11	7 partner, 3 daughter/son, 2 mother/father, 1 sister/brother, and 1 other degree of relatedness	(18)	3

U = Unspecified.

## Data Availability

No new data were created or analyzed in this study. Data sharing is not applicable to this article.

## Supplementary Materials

The following supporting information can be downloaded at: https://www.mdpi.com/article/10.3390/nursrep14020113/s1, Table S1: Coding tree.

## Public Involvement Statement

No public involvement in any aspect of this research.

## Guidelines and Standards Statement

This manuscript was drafted following the Preferred Reporting Items for Systematic Reviews and Meta-Analyses (PRISMA) Statement.
