# Supplementary material for: The Experiences of Family Members of Patients Discharged from Intensive Care Unit: A Systematic Review of Qualitative Studies
Source: Nurs Rep. 2024 Jun 14;14(2):1504–16. doi: 10.3390/nursrep14020113 (PMC11206407; doi:10.3390/nursrep14020113)
Supplement: Supplementary file 1 [file nursrep-14-00113-s001.zip › nursrep-3019925-supplementary.pdf]

Supplementary Table S1. Coding tree.

Abstraction: themes	Abstraction: categories	Codes	Example from quotes
**Grappling with a weighty burden**	**Weight of responsibility**	Lots of duties	*"Suddenly I got a lot of responsibilities, things I had never tried and I just didn't know what to do."* (Ågård et al., 2015)<br><br>*"Fear that I pass away, but the real fear is: how will my wife save herself then? As a matter of fact, she can't miss [function without] me."* (Van Sleeuwen et al., 2020)
		Filling different roles	*"I have a different role here. I have to fill many roles at the same time and be 'husband and wife, caregiver or lover,' or whatever we call it…"* (Ågård et al., 2015)<br><br>*"Sometimes it just looks like I'm the mother and he's the kid …"* (Van Sleeuwen et al., 2020)<br><br>*"….I'm the home maid, the cleaner, the dishwasher, the cook, the one who buys the groceries"* (Nelderup et al.,2020)
		Difficulties in social relations	*"Both family and friends have been very good ….But now as time goes on we don't have the energy to return their favors"* (Ågård et al., 2015)<br><br>*"After this happened to my husband, I had a fight with a lot of people, as I thought they interfered with things they shouldn't interfere with."* (Van Sleeuwen et al., 2020)<br><br>*"I was not [comfortable going] out in public. I had tried a couple of times at the store, but then someone came to me, people approached me, and I could not handle that they had asked me about things. . ."* (Frivold et al., 2016)
	**Maladaptation to caregiving role**	Feeling overwhelmed	"*It takes so much time. I do it every time. I had to go to the airport the other day, you know. Then we need some milk. Then the car needs a check-up. I have to go again ... every time. Food ... I have to shop and cook each day."* (Ågård et al., 2015)<br><br>*".. Everything was up to me and it was really a burden"* (Ågård et al., 2015)<br><br>*"Oh I had no idea what I was in for [when my son came home]… I don't think I slept two hours the first week. That's up and down the stairs, he was so much work"* (Czerwonka et al., 2015)<br><br>*I have started wondering if I have become senile. . .my wording, when I am going to express myself I suddenly feel that I can't find the right words....and that I relate [those feelings] to these experiences [of my husband's illness]"* (Frivold et al., 2016)<br><br>*"…– I just don't have the energy, because there are so many loose ends here"* (Ågård et al., 2015)<br><br>*"I'm so busy, sometimes I think I can't keep going, I'm stuck…."* (Nelderup et al.,2020)

			"It seems I'm worse tired now than I was when she was sick and right out the hospital. I don't know whether it's just catching up with me or not, but I'm mentally and emotionally exhausted ... Everything just seems like a struggle lately." (Choi et al., 2018)<br><br>*"I didn't want to eat, I didn't want to live. I thought: if he's gone, I have to be gone."* (Van Sleeuwen et al., 2020)<br><br>*"I'm so busy, sometimes I think I can't keep going, I'm stuck. Shall I tell him [the patient]? He probably has something else on his mind."* (Van Sleeuwen et al., 2020)
		Feeling lost	*"It seems I'm worse tired now than I was when she was sick and right out the hospital. I don't know whether it's just catching up with me or not, but I'm mentally and emotionally exhausted ... Everything just seems like a struggle lately."* (Choi et al., 2018)<br><br>*"It's easy to make mistakes and nobody else was there…."* (Ågård et al., 2015)<br><br>"*Then I was* ***scared*** *to death that he was coming home. No, I said! Never in my life. I can't manage that…"* (Nelderup et al.,2020)<br><br>*"Left without a lifeline"* (Vester et al.,2022)
		Feeling angry	*"I was so* ***angry*** *that I cried."* (Ågård et al., 2015)
		Feeling worried	*"It's still a* ***worry****-when I wake up in the morning, the first thing I do is see if she's alright…"* (Czerwonka et al., 2015)<br><br>*"...I was on my guard and every time there was a thud I thought that he'd fallen. He had a walker but I was still on tenterhooks but I'm not anymore. He's as he used to be"* (Nelderup et al.,2020)<br><br>*"Coming home is a little scary to me. I worry about taking care of him... what it's gonna involve."* (Choi et al., 2018)
		Feeling frustrate	*"Being negative about everything. Passing judgements on everything, everything was foolish"* (Nelderup et al.,2020)<br><br>*"It was* ***frustrating*** *to discover that things got lost in the system. I discovered that there were other options, but they were dead ends as well"* (Ågård et al., 2015)<br><br>*"No one can tell us how to increase that activity level appropriately; it's really just trial and error, so it's a little frustrating."* (Choi et al., 2018)
**Recognizing and confronting adversities**	**Challenges encountered**	Managing medical complexities	*things I had never tried and I just didn't know what to do."* (Ågård et al., 2015)<br><br>*"That day I just didn't know what to do…. I finally thought: 'What should I do? I am not a*

**along the way**			*nurse.'* (Ågård et al., 2015)<br><br>*"I received quite a lot of information; I think mainly because I was very persistent. I was always there asking questions and then if they told me anything, I basically went back to the computer and researched it[...] because the information that was passed on to me was, at that time, too much to take in because I had no medical knowledge."* (Czerwonka et al., 2015)
		Too much caring work	*"...I can't manage that and what if he falls to the floor and if he doesn't obey and starts to argue about what I've decided and you know with the medication and everything" "* (Nelderup et al.,2020)<br><br>*"I had to take time of work to assist her'"* (Danielis et al., 2022)<br><br>*That's up and down the stairs, he was so much work"* (Czerwonka et al., 2015)
		Letting things go at their pace	*We have only each other to consider and can doing things at our own pace."* (Ågård et al., 2015)<br><br>*"You lean back and say: Okay, it will just have to go as it goes. You just give up and let things go at their pace."* (Ågård et al., 2015)<br><br>*"Everything is going smoothly. The calendar is fairly well-filled. We're doing things as before. Life goes on"* (Nelderup et al.,2020)<br><br>*"The experience is part of who I am; in this way, I will continue my life"* (Vester et al.,2022)
		Having no idea of the patient's prognosis	*"Is it true, he's getting better?"* (Vester et al., 2022)<br><br>*"No one seems to know how long his condition is going to be the way it is or if it is ever going to be any different, if he's ever going to get better, or if he's just going to stay the same."* (Choi et al., 2018)<br><br>*"I just hope the lung infection doesn't come back again…he wouldn't survive another illness"* (Danielis et al., 2022)
	**Ability to cope with the demanding situation**	Feeling strong	*"I have managed well through it, and I work as usual, and I have no physical or emotional effects at this point."* (Frivold et al., 2016)<br><br>*"I have been good at handling things myself, and of course having my children around all the time. So I had someone to talk to. But not too much, I felt I had to do it myself... so I have been working on that. . ."* (Frivold et al., 2016)<br><br>*"I am not used to asking for help. You manage on your own. I discovered an inner strength, and I am stronger than I thought"* (Frivold et al., 2016)

		Recognizing important values	*"Maybe it [my life] has gained another dimension, making it better."* (Nelderup et al.,2020)<br><br>*"This is how life is. It has a beginning and it has an end. And you can't make the end worse than it has to be. Instead you have to think back to the good things we have experienced together, all the trips we have made. That's how you need to think"* (Nelderup et al.,2020)<br><br>*"So, I feel grateful, but it doesn't seem like my husband does. At the same time, I admit that he didn't know how ill he actually was...so I think: You have received life as a gift, utilise it, do something, learn something new, and get out"* (Frivold et al., 2016)<br><br>*"...from that day all my focus was on him. So and...I put all focus on that. And that's what you have to pay for later, but I was still happy that I did as I did"* (Nelderup et al.,2020)<br><br>*"I want us to be like before. I hope we will.."* (Vester et al.,2022)<br><br>*"No matter how beautiful we live in this lovely, thatched farmhouse with a marvellous swimming pool, it is not worth it to me."* (Nelderup et al.,2020)<br><br>*"I didn't have anything to do with the church. However, I've been thinking differently about this. It helped me in a positive way."* (Nelderup et al.,2020)<br><br>*"Life-threatening experiences changes your perspective. The friends who stick around and listen, you hold onto. Those who don't, you end"* (Vester et al.,2022)<br><br>*"...thus you end up neglecting much of your own feelings; what is bothering you is neglected. I have put it away, and I have to deal with it little by little"* (Frivold et al., 2016)
**Seeking supports beyond one's own resources**	**Peer-based and professional supports**	Support given by the healthcare system<br><br><br><br><br><br><br><br><br><br>Unsupportive healthcare system	*"The home care nurse, once he was discharged, she came right over the next day, she was phenomenal. ... she was just tremendous. She just walked us through everything. I felt really good while she was there, and after she left I knew everything would be okay."* (Czerwonka et al., 2015)<br><br>*"It has given me incredible joy to see such a good side of the health care system"* (Frivold et al., 2016)<br><br>*"At home, we are followed by home care, a nurse visits us. If there are any critical issues, we ask her"* (Danielis et al., 2022)<br><br>*"I feel that there was about two week delay for his rehabilitation to start. And the reason I'm emphasizing on the delay is because two weeks after an ICU stay for a survivor is a long, long time."* (Czerwonka et al., 2015)<br><br>*"So I call and I call. . . [and they answer the phone]. "Oh, yes, you haven't got an appointment for a return visit we will arrange that". So, it's maybe a week or a month, and*

			still no information comes. . . so I call again" (Frivold et al., 2016)<br><br>*"I am not more worried or anything, but of course my confidence in the healthcare system, and this ICU in particular, is gone"* (Frivold et al., 2016)
		Support provided by friends and relatives.	*"Both family and friends have been very good at visiting, writing and calling."* (Ågård et al., 2015)<br><br>*"Listening to people sharing their struggles with everyday life validated my experiences. They were finally real!"* (Vester et al.,2022)<br><br>*I have support from my children, but, of course, they have jobs." (Choi et al., 2018)*
	**Essential needs for caregiving**	Needs for assistance	*If I had only had some instructions or a contact person or someone who said: 'Listen, I will take care of this."* (Ågård et al., 2015)<br><br>*I finally called the doctor and asked him to come as fast as possible."* (Ågård et al., 2015)<br><br>*"I think [1] probably [would have liked to receive more contact] with the health care system, because you're not quite sure as to how your recovery period is going."* (Czerwonka et al., 2015)<br><br>*"As to rehabilitation, I opted for a private service. I called a physiotherapist who is now helping us"* (Danielis et al., 2022)<br><br>*".....there isn't someone managing the whole thing, managing all the components of his life... like how to fully recover.[.) there should be someone managing the whole case... there should have been the suggestion that we go into a rehab situation... I think things like that could have been managed better." ."* (Czerwonka et al., 2015)
		Psychological needs	*"And those psychologists told us to have some drawers to close sometimes. You can't let it all out at once."* (Frivold et al., 2016)<br><br>*"Actually, I considered whether I needed help from a psychologist to clear up my thoughts, [so] that I'm not as mean when I'm upset"* (Frivold et al., 2016)<br><br>*"They recommended that she see a therapist and we thought that was dumb. But now I wished she would have done it earlier.... I think she is even more depressed"* (Choi et al.,2018)
		Setting the home environment	*"I got a stationary bike to allow him to do some exercise. He cannot still climb the stairs"* (Danielis et al., 2022)<br><br>*"It was a little bit hard in the beginning because I had to renovate and change some things at home. Put in some technical aids. Our old house is definitely not suited for a handicapped person…"* (Nelderup et al.,2020)

**Targeting care provision**	**Activities performed as caregiver**	Continuous monitoring	*"The three of us in the family are home and observe her all the time; if she has a bad day, worse than usual, we are much more alert."* (Ågård et al., 2015)<br><br>*"… the first thing I do is see if she's alright, I go across to the bed and she's laying there sound asleep so then we get up"* (Czerwonka et al., 2015)<br><br>*"When we got home, I used to sleep in the living room so as not to stay too close and bring bacteria or viruses near her"* (Danielis et al., 2022)<br><br>*"I also have to get someone to do grocery shopping so that I don't leave her alone"* (Danielis et al., 2022)
		Supporting the patient	*"I couldn't just sit and watch how he had fought at the hospital and then just faded away. So, I said that either he would pull himself together and take a walk, or I would go to the summer house and he would have to manage on his own.* (Ågård et al., 2015)<br><br>*"My main job right now is to help him pace himself appropriately and to help him when he doesn't."*(Choi et al., 2018)
		Talking about the disease path with the patient	*"That was probably the hardest thing for me though; just explaining to her like having tubes down her throat and being in a coma for like three weeks. But, to be honest, I was surprised how much she wanted to know."* (Choi et al., 2018)<br><br>*"I don't want to be a nag, but I want to try to give him a realistic picture of where he is at and where he is going... I am glad he listens and hopefully that will help prevent him from having unrealistic expectations and getting frustrated as he goes along over the next few months."* (Choi et al., 2018)<br><br>*"I don't want to remind him how bad he was .... I find it hard for him to talk about it sometimes. I don't know if it makes things worse when we talk about it because it reminds him of how weak he was."* (Choi et al., 2018)<br><br>*"Actually, it was very good to sit there, talking about it, and he [the patient] got answers to questions that, even if I had tried to explain to him, [I couldn't have given him] and we had talked about the events, medical treatments, and so on. Now, he has the exact answers"* (Frivold et al., 2016)
	**Patient' dependency**	Patient's participation	*"I couldn't get him to go to rehab. He just sat on the couch and stared out the window…. But then he got up and walked to the beach and back. The day after, he came along with me twice"* (Ågård et al., 2015)<br><br>*"…and what I find the most difficult, is getting (my husband) to take ownership of this.... I get a little impatient because I think if you're the survivor, you've got to take ownership for your well being... It's not something that I can make him do [exercise], he has to want to do it, but he also has to feel well enough that he wants to do it. And I think that's part of the problem - that he just doesn't feel well."* (Czerwonka et al., 2015)

			The most difficult thing is seeing him not being able to deal with it. He just breaks down, and he just sits there and cries because he does not know what to do. And it is sort of hard to see him when I know he is such a strong guy." (Choi et al.,2018)
		Long-term care and concerns	*"I was afraid that he would become depressed. Nothing happened. After 3 months there was no progress"* (Ågård et al., 2015)<br><br>*"... [My husband's) body was so stiff from being bed ridden for so many months and his weight was reduced about 80 pounds. He needed to learn to walk and go on with his motor skills."* (Czerwonka et al., 2015)<br><br>*"He doesn't walk, he doesn't move by himself, he can't even sign."* (Danielis et al., 2022)<br><br>*"He says that he hasn't suffered permanent damage, but it just isn't true. He has lots of problems, but not physical. I mean, invisible things that have changed."* (Ågård et al., 2015)<br><br>*".. Like he can go out and do something one day, feel pretty good, but then 2 days later he'll be really tired."* (Choi et al.,2018)
